# Dental Legislation

**Published:** 1879-05

**Authors:** 


### DENTAL LEGISLATION.

Below is given the text of a bill now pending in the legislature of Michigan, for the regulation of the practice of dentistry in that state. The bill is, in most respects, an excellent one.

It is not retroactive—does not affect any who are now in practice in the state; but those outside of the state, who after the passage of the law seek to enter the practice in the state, are subject to its restrictions, and any who, after its passage, begin practice are amenable to it.

The registration feature is good, the law of no other state, except Kentucky, has such a requirement, all should have it. The penalty has an imprisonment clause, in this respect making it more effective than the dental law of any other state.

There is little doubt but that the bill will become a law; and if so the dental profession of Michigan will stand in the forefront in respect to progress.

It will have the best dental law in the country, and one of the best dental colleges in the world. Every state should have a similar law, and the profession should see that it is obeyed.

A BILL

*To regulate the Practice of Dentistry.*

SECTION 1. *The people of the State of Michigan enact*, That it shall be unlawful for any person to practice dentistry in the State of Michigan, for compensation, unless such person has been in the regular practice of dentistry within this state previous to the passage of this act, or has received a diploma from the faculty of a dental college duly incorporated under the laws of this or any other state of the United States, or a certificate of qualification issued by the authority of a board of censors appointed by the governor of the State of Michigan.

SEC. 2. It shall be the duty of the governor, upon the passage of this act, to appoint a board of censors, consisting of not less than five persons, whose duty it shall be to ascertain the qualifications of each and every applicant, which examination being satisfactory, a certificate shall be issued to said applicant or applicants, signed by a majority of the members of said board of censors, and the person so licensed shall pay into the treasury of said board the sum of ten dollar to defray the expenses of said board. The said board of censors shall qualify and organize within thirty days after their appointment, by the election of a president, secretary and treasurer, and shall meet thereafter semi-annually as follows: On the second Tuesday of June, in Grand Rapids, and on the second Tuesday in January, in Detroit, for the examination of all applicants, and at such other places as the board may from time to time direct: *Provided*, That at least one month's notice shall be given of such meetings for examinations. Each member of said board shall be entitled to five dollars per day, and five cents milage fee, not to exceed three days at any one session: *Provided*, all the expenses of the board of examiners shall be paid from the funds received by said board from examination fees. The manner of appointment shall be: one for five years, one for four years, one for three years, one for two years, one for one year, and one for one year, each year thereafter.

Sec. 3. The said board of censors shall cause to be kept a book in which shall be registered the names of all practitioners of dentistry in the State of Michigan who are authorized to practice by the provisions of this act, and the book or books so kept shall be a book or books of record, and a transcript from the same, certified by the officer who has it in charge, with the signature of the officers of said board affixed thereto, shall be evidence in any court in this state.

Sec. 4. Any person who shall, in violation of this act, practice dentistry in the State of Michigan, shall be deemed guilty of a misdemeanor and upon conviction thereof shall be fined not less than twenty-five dollars nor more than one hundred dollars, or imprisonment in the county jail not less than five nor more than thirty days, or both, in the discretion of the court, for each and every offense: *Provided*, That nothing in this act shall be construed to prevent any person from extracting teeth, and making artificial teeth.

Sec. 5. All prosecutions under this act shall be by complaint or information before any court of competent jurisdiction, in the county where the offense is committed, and all fines imposed and collected under the provisions of this act, shall be paid into the treasury of the county where such conviction shall take place, for the use of the primary school fund within such county.

Sec. 6. Every person holding a certificate from a board of examiners shall have it recorded in the office of the clerk of the county in which he resides, and the record shall be endorsed thereon. Any person removing to another county to practice shall procure an endorsement to that effect on the certificate from the county clerk, and shall record the certificate in like manner in the county to which he or she removes, and the holder of the certificate shall pay to the county clerk the usual fee for making the record.

